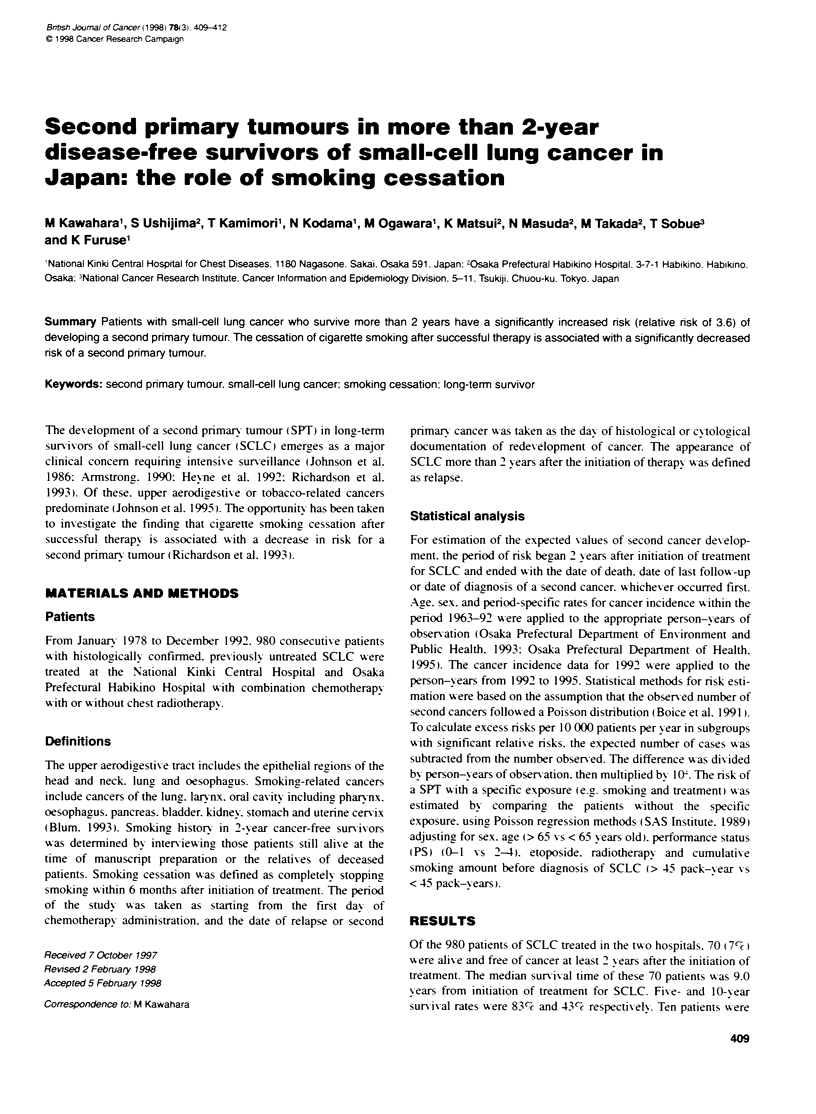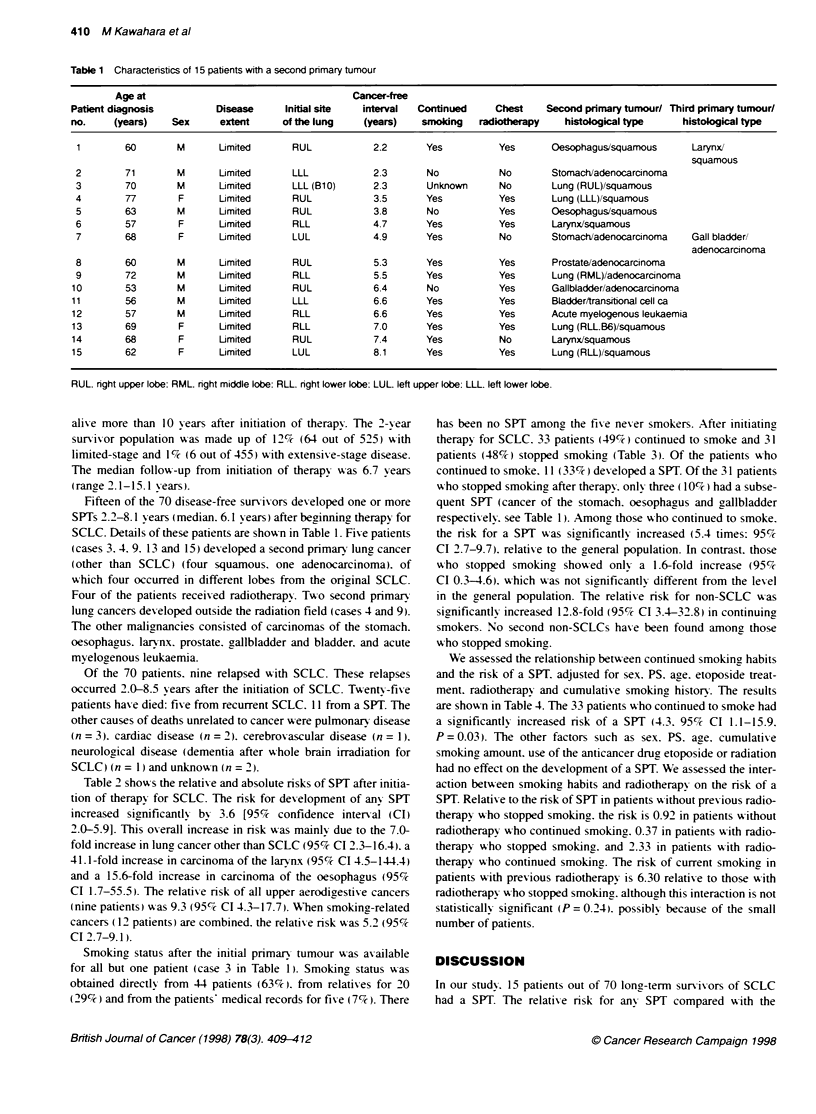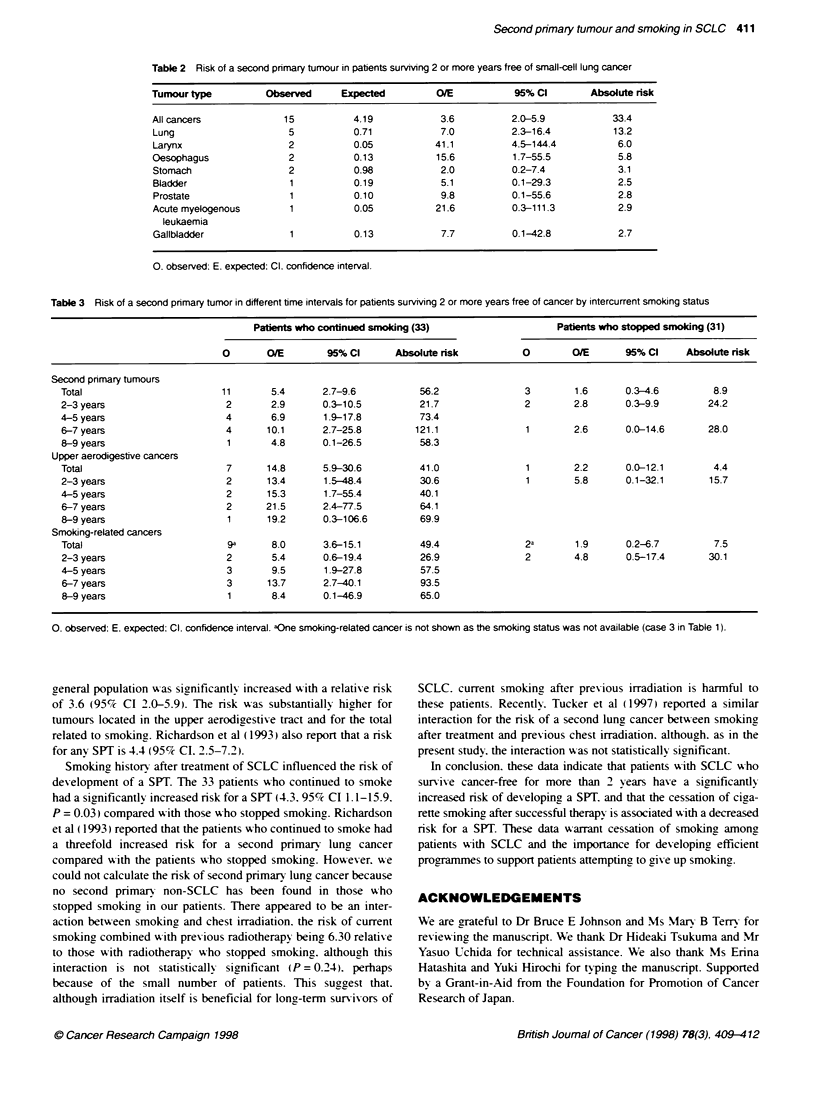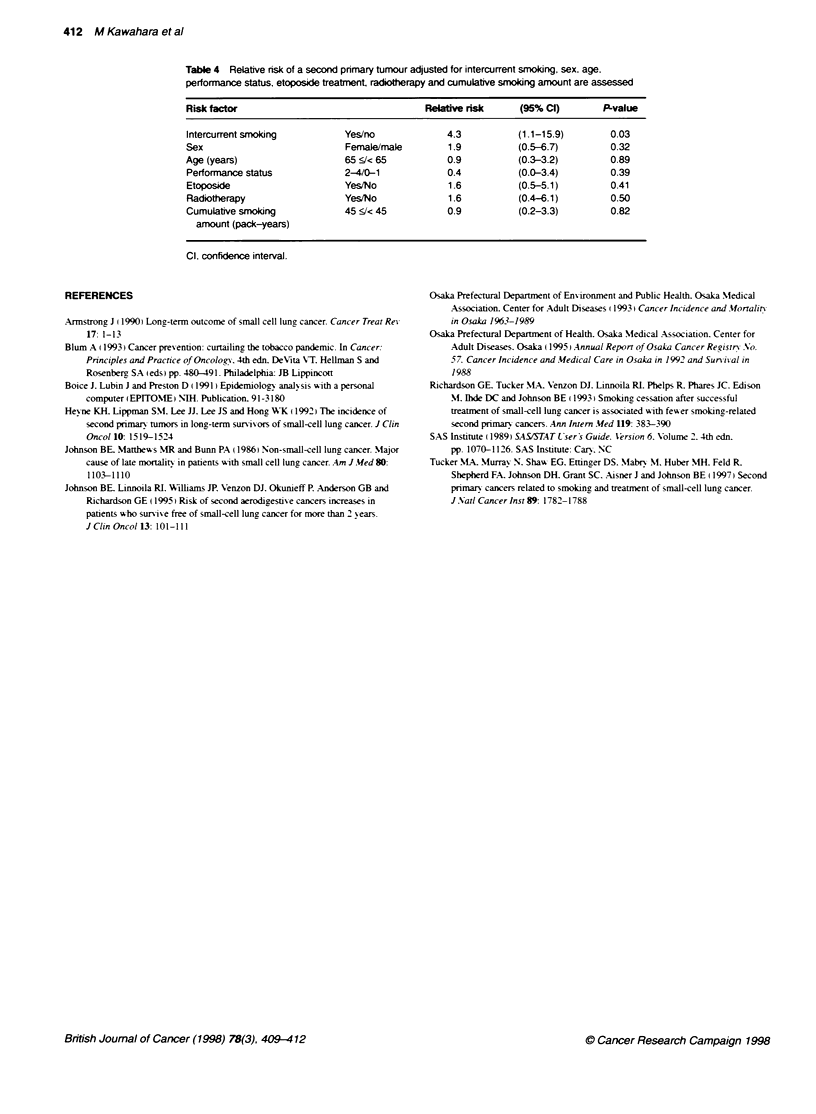# Second primary tumours in more than 2-year disease-free survivors of small-cell lung cancer in Japan: the role of smoking cessation.

**DOI:** 10.1038/bjc.1998.507

**Published:** 1998-08

**Authors:** M. Kawahara, S. Ushijima, T. Kamimori, N. Kodama, M. Ogawara, K. Matsui, N. Masuda, M. Takada, T. Sobue, K. Furuse

**Affiliations:** National Kinki Central Hospital for Chest Diseases, Sakai, Osaka, Japan.

## Abstract

Patients with small-cell lung cancer who survive more than 2 years have a significantly increased risk (relative risk of 3.6) of developing a second primary tumour. The cessation of cigarette smoking after successful therapy is associated with a significantly decreased risk of a second primary tumour.


British Journal of Cancer (1998) 78(3), 409–412
© 1998 Cancer Research Campaign

### Second primary tumours in more than 2-year disease-free survivors of small-cell lung cancer in Japan: the role of smoking cessation

**M Kawahara[1], S Ushijima[2], T Kamimori[1], N Kodama[1], M Ogawara[1], K Matsui[2], N Masuda[2], M Takada[2], T Sobue[3] and K Furuse[1]**

[1]National Kinki Central Hospital for Chest Diseases, 1180 Nagasone, Sakai, Osaka 591, Japan; [2]Osaka Prefectural Habikino Hospital, 3-7-1 Habikino, Habikino, Osaka; [3]National Cancer Research Institute, Cancer Information and Epidemiology Division, 5–11, Tsukiji, Chuou-ku, Tokyo, Japan

**Summary** Patients with small-cell lung cancer who survive more than 2 years have a significantly increased risk (relative risk of 3.6) of developing a second primary tumour. The cessation of cigarette smoking after successful therapy is associated with a significantly decreased risk of a second primary tumour.

**Keywords:** second primary tumour; small-cell lung cancer; smoking cessation; long-term survivor

The development of a second primary tumour (SPT) in long-term survivors of small-cell lung cancer (SCLC) emerges as a major clinical concern requiring intensive surveillance (Johnson et al, 1986; Armstrong, 1990; Heyne et al, 1992; Richardson et al, 1993). Of these, upper aerodigestive or tobacco-related cancers predominate (Johnson et al, 1995). The opportunity has been taken to investigate the finding that cigarette smoking cessation after successful therapy is associated with a decrease in risk for a second primary tumour (Richardson et al, 1993).

### MATERIALS AND METHODS

### Patients

From January 1978 to December 1992, 980 consecutive patients with histologically confirmed, previously untreated SCLC were treated at the National Kinki Central Hospital and Osaka Prefectural Habikino Hospital with combination chemotherapy with or without chest radiotherapy.

### Definitions

The upper aerodigestive tract includes the epithelial regions of the head and neck, lung and oesophagus. Smoking-related cancers include cancers of the lung, larynx, oral cavity including pharynx, oesophagus, pancreas, bladder, kidney, stomach and uterine cervix (Blum, 1993). Smoking history in 2-year cancer-free survivors was determined by interviewing those patients still alive at the time of manuscript preparation or the relatives of deceased patients. Smoking cessation was defined as completely stopping smoking within 6 months after initiation of treatment. The period of the study was taken as starting from the first day of chemotherapy administration, and the date of relapse or second primary cancer was taken as the day of histological or cytological documentation of redevelopment of cancer. The appearance of SCLC more than 2 years after the initiation of therapy was defined as relapse.

*Received 7 October 1997*
*Revised 2 February 1998*
*Accepted 5 February 1998*

*Correspondence to:* M Kawahara

### Statistical analysis

For estimation of the expected values of second cancer development, the period of risk began 2 years after initiation of treatment for SCLC and ended with the date of death, date of last follow-up or date of diagnosis of a second cancer, whichever occurred first. Age, sex, and period-specific rates for cancer incidence within the period 1963–92 were applied to the appropriate person–years of observation (Osaka Prefectural Department of Environment and Public Health, 1993; Osaka Prefectural Department of Health, 1995). The cancer incidence data for 1992 were applied to the person–years from 1992 to 1995. Statistical methods for risk estimation were based on the assumption that the observed number of second cancers followed a Poisson distribution (Boice et al, 1991). To calculate excess risks per 10 000 patients per year in subgroups with significant relative risks, the expected number of cases was subtracted from the number observed. The difference was divided by person–years of observation, then multiplied by $10^4$. The risk of a SPT with a specific exposure (e.g. smoking and treatment) was estimated by comparing the patients without the specific exposure, using Poisson regression methods (SAS Institute, 1989) adjusting for sex, age (> 65 vs < 65 years old), performance status (PS) (0–1 vs 2–4), etoposide, radiotherapy and cumulative smoking amount before diagnosis of SCLC (> 45 pack–year vs < 45 pack–years).

### RESULTS

Of the 980 patients of SCLC treated in the two hospitals, 70 (7%) were alive and free of cancer at least 2 years after the initiation of treatment. The median survival time of these 70 patients was 9.0 years from initiation of treatment for SCLC. Five- and 10-year survival rates were 83% and 43% respectively. Ten patients were

**Table 1** Characteristics of 15 patients with a second primary tumour

| Patient no. | Age at diagnosis (years) | Sex | Disease extent | Initial site of the lung | Cancer-free interval (years) | Continued smoking | Chest radiotherapy | Second primary tumour/ histological type | Third primary tumour/ histological type |
|---|---|---|---|---|---|---|---|---|---|
| 1 | 60 | M | Limited | RUL | 2.2 | Yes | Yes | Oesophagus/squamous | Larynx/ squamous |
| 2 | 71 | M | Limited | LLL | 2.3 | No | No | Stomach/adenocarcinoma | |
| 3 | 70 | M | Limited | LLL (B10) | 2.3 | Unknown | No | Lung (RUL)/squamous | |
| 4 | 77 | F | Limited | RUL | 3.5 | Yes | Yes | Lung (LLL)/squamous | |
| 5 | 63 | M | Limited | RUL | 3.8 | No | Yes | Oesophagus/squamous | |
| 6 | 57 | F | Limited | RLL | 4.7 | Yes | Yes | Larynx/squamous | |
| 7 | 68 | F | Limited | LUL | 4.9 | Yes | No | Stomach/adenocarcinoma | Gall bladder/ adenocarcinoma |
| 8 | 60 | M | Limited | RUL | 5.3 | Yes | Yes | Prostate/adenocarcinoma | |
| 9 | 72 | M | Limited | RLL | 5.5 | Yes | Yes | Lung (RML)/adenocarcinoma | |
| 10 | 53 | M | Limited | RUL | 6.4 | No | Yes | Gallbladder/adenocarcinoma | |
| 11 | 56 | M | Limited | LLL | 6.6 | Yes | Yes | Bladder/transitional cell ca | |
| 12 | 57 | M | Limited | RLL | 6.6 | Yes | Yes | Acute myelogenous leukaemia | |
| 13 | 69 | F | Limited | RLL | 7.0 | Yes | Yes | Lung (RLL.B6)/squamous | |
| 14 | 68 | F | Limited | RUL | 7.4 | Yes | No | Larynx/squamous | |
| 15 | 62 | F | Limited | LUL | 8.1 | Yes | Yes | Lung (RLL)/squamous | |

RUL. right upper lobe: RML. right middle lobe: RLL. right lower lobe: LUL. left upper lobe: LLL. left lower lobe.

alive more than 10 years after initiation of therapy. The 2-year survivor population was made up of 12% (64 out of 525) with limited-stage and 1% (6 out of 455) with extensive-stage disease. The median follow-up from initiation of therapy was 6.7 years (range 2.1–15.1 years).

Fifteen of the 70 disease-free survivors developed one or more SPTs 2.2–8.1 years (median. 6.1 years) after beginning therapy for SCLC. Details of these patients are shown in Table 1. Five patients (cases 3. 4. 9. 13 and 15) developed a second primary lung cancer (other than SCLC) (four squamous. one adenocarcinoma). of which four occurred in different lobes from the original SCLC. Four of the patients received radiotherapy. Two second primary lung cancers developed outside the radiation field (cases 4 and 9). The other malignancies consisted of carcinomas of the stomach. oesophagus. larynx. prostate. gallbladder and bladder. and acute myelogenous leukaemia.

Of the 70 patients. nine relapsed with SCLC. These relapses occurred 2.0–8.5 years after the initiation of SCLC. Twenty-five patients have died: five from recurrent SCLC. 11 from a SPT. The other causes of deaths unrelated to cancer were pulmonary disease ($n = 3$). cardiac disease ($n = 2$). cerebrovascular disease ($n = 1$). neurological disease (dementia after whole brain irradiation for SCLC) ($n = 1$) and unknown ($n = 2$).

Table 2 shows the relative and absolute risks of SPT after initiation of therapy for SCLC. The risk for development of any SPT increased significantly by 3.6 [95% confidence interval (CI) 2.0–5.9]. This overall increase in risk was mainly due to the 7.0-fold increase in lung cancer other than SCLC (95% CI 2.3–16.4). a 41.1-fold increase in carcinoma of the larynx (95% CI 4.5–144.4) and a 15.6-fold increase in carcinoma of the oesophagus (95% CI 1.7–55.5). The relative risk of all upper aerodigestive cancers (nine patients) was 9.3 (95% CI 4.3–17.7). When smoking-related cancers (12 patients) are combined. the relative risk was 5.2 (95% CI 2.7–9.1).

Smoking status after the initial primary tumour was available for all but one patient (case 3 in Table 1). Smoking status was obtained directly from 44 patients (63%). from relatives for 20 (29%) and from the patients' medical records for five (7%). There has been no SPT among the five never smokers. After initiating therapy for SCLC. 33 patients (49%) continued to smoke and 31 patients (48%) stopped smoking (Table 3). Of the patients who continued to smoke. 11 (33%) developed a SPT. Of the 31 patients who stopped smoking after therapy. only three (10%) had a subsequent SPT (cancer of the stomach. oesophagus and gallbladder respectively. see Table 1). Among those who continued to smoke. the risk for a SPT was significantly increased (5.4 times: 95% CI 2.7–9.7). relative to the general population. In contrast. those who stopped smoking showed only a 1.6-fold increase (95% CI 0.3–4.6). which was not significantly different from the level in the general population. The relative risk for non-SCLC was significantly increased 12.8-fold (95% CI 3.4–32.8) in continuing smokers. No second non-SCLCs have been found among those who stopped smoking.

We assessed the relationship between continued smoking habits and the risk of a SPT. adjusted for sex. PS. age. etoposide treatment. radiotherapy and cumulative smoking history. The results are shown in Table 4. The 33 patients who continued to smoke had a significantly increased risk of a SPT (4.3. 95% CI 1.1–15.9. $P = 0.03$). The other factors such as sex. PS. age. cumulative smoking amount. use of the anticancer drug etoposide or radiation had no effect on the development of a SPT. We assessed the interaction between smoking habits and radiotherapy on the risk of a SPT. Relative to the risk of SPT in patients without previous radiotherapy who stopped smoking. the risk is 0.92 in patients without radiotherapy who continued smoking. 0.37 in patients with radiotherapy who stopped smoking. and 2.33 in patients with radiotherapy who continued smoking. The risk of current smoking in patients with previous radiotherapy is 6.30 relative to those with radiotherapy who stopped smoking. although this interaction is not statistically significant ($P = 0.24$). possibly because of the small number of patients.

### DISCUSSION

In our study. 15 patients out of 70 long-term survivors of SCLC had a SPT. The relative risk for any SPT compared with the

© Cancer Research Campaign 1998

**Table 2** Risk of a second primary tumour in patients surviving 2 or more years free of small-cell lung cancer

| Tumour type | Observed | Expected | O/E | 95% CI | Absolute risk |
|---|---|---|---|---|---|
| All cancers | 15 | 4.19 | 3.6 | 2.0–5.9 | 33.4 |
| Lung | 5 | 0.71 | 7.0 | 2.3–16.4 | 13.2 |
| Larynx | 2 | 0.05 | 41.1 | 4.5–144.4 | 6.0 |
| Oesophagus | 2 | 0.13 | 15.6 | 1.7–55.5 | 5.8 |
| Stomach | 2 | 0.98 | 2.0 | 0.2–7.4 | 3.1 |
| Bladder | 1 | 0.19 | 5.1 | 0.1–29.3 | 2.5 |
| Prostate | 1 | 0.10 | 9.8 | 0.1–55.6 | 2.8 |
| Acute myelogenous leukaemia | 1 | 0.05 | 21.6 | 0.3–111.3 | 2.9 |
| Gallbladder | 1 | 0.13 | 7.7 | 0.1–42.8 | 2.7 |

O. observed: E. expected: CI. confidence interval.

**Table 3** Risk of a second primary tumor in different time intervals for patients surviving 2 or more years free of cancer by intercurrent smoking status

| | Patients who continued smoking (33) | | | | Patients who stopped smoking (31) | | | |
|---|---|---|---|---|---|---|---|---|
| | O | O/E | 95% CI | Absolute risk | O | O/E | 95% CI | Absolute risk |
| Second primary tumours | | | | | | | | |
| Total | 11 | 5.4 | 2.7–9.6 | 56.2 | 3 | 1.6 | 0.3–4.6 | 8.9 |
| 2–3 years | 2 | 2.9 | 0.3–10.5 | 21.7 | 2 | 2.8 | 0.3–9.9 | 24.2 |
| 4–5 years | 4 | 6.9 | 1.9–17.8 | 73.4 | | | | |
| 6–7 years | 4 | 10.1 | 2.7–25.8 | 121.1 | 1 | 2.6 | 0.0–14.6 | 28.0 |
| 8–9 years | 1 | 4.8 | 0.1–26.5 | 58.3 | | | | |
| Upper aerodigestive cancers | | | | | | | | |
| Total | 7 | 14.8 | 5.9–30.6 | 41.0 | 1 | 2.2 | 0.0–12.1 | 4.4 |
| 2–3 years | 2 | 13.4 | 1.5–48.4 | 30.6 | 1 | 5.8 | 0.1–32.1 | 15.7 |
| 4–5 years | 2 | 15.3 | 1.7–55.4 | 40.1 | | | | |
| 6–7 years | 2 | 21.5 | 2.4–77.5 | 64.1 | | | | |
| 8–9 years | 1 | 19.2 | 0.3–106.6 | 69.9 | | | | |
| Smoking-related cancers | | | | | | | | |
| Total | 9[a] | 8.0 | 3.6–15.1 | 49.4 | 2[a] | 1.9 | 0.2–6.7 | 7.5 |
| 2–3 years | 2 | 5.4 | 0.6–19.4 | 26.9 | 2 | 4.8 | 0.5–17.4 | 30.1 |
| 4–5 years | 3 | 9.5 | 1.9–27.8 | 57.5 | | | | |
| 6–7 years | 3 | 13.7 | 2.7–40.1 | 93.5 | | | | |
| 8–9 years | 1 | 8.4 | 0.1–46.9 | 65.0 | | | | |

O. observed: E. expected: CI. confidence interval. [a]One smoking-related cancer is not shown as the smoking status was not available (case 3 in Table 1).

general population was significantly increased with a relative risk of 3.6 (95% CI 2.0–5.9). The risk was substantially higher for tumours located in the upper aerodigestive tract and for the total related to smoking. Richardson et al (1993) also report that a risk for any SPT is 4.4 (95% CI. 2.5–7.2).

Smoking history after treatment of SCLC influenced the risk of development of a SPT. The 33 patients who continued to smoke had a significantly increased risk for a SPT (4.3. 95% CI 1.1–15.9. $P = 0.03$) compared with those who stopped smoking. Richardson et al (1993) reported that the patients who continued to smoke had a threefold increased risk for a second primary lung cancer compared with the patients who stopped smoking. However. we could not calculate the risk of second primary lung cancer because no second primary non-SCLC has been found in those who stopped smoking in our patients. There appeared to be an interaction between smoking and chest irradiation. the risk of current smoking combined with previous radiotherapy being 6.30 relative to those with radiotherapy who stopped smoking. although this interaction is not statistically significant ($P = 0.24$). perhaps because of the small number of patients. This suggest that. although irradiation itself is beneficial for long-term survivors of SCLC. current smoking after previous irradiation is harmful to these patients. Recently. Tucker et al (1997) reported a similar interaction for the risk of a second lung cancer between smoking after treatment and previous chest irradiation. although. as in the present study. the interaction was not statistically significant.

In conclusion. these data indicate that patients with SCLC who survive cancer-free for more than 2 years have a significantly increased risk of developing a SPT. and that the cessation of cigarette smoking after successful therapy is associated with a decreased risk for a SPT. These data warrant cessation of smoking among patients with SCLC and the importance for developing efficient programmes to support patients attempting to give up smoking.

### ACKNOWLEDGEMENTS

We are grateful to Dr Bruce E Johnson and Ms Mary B Terry for reviewing the manuscript. We thank Dr Hideaki Tsukuma and Mr Yasuo Uchida for technical assistance. We also thank Ms Erina Hatashita and Yuki Hirochi for typing the manuscript. Supported by a Grant-in-Aid from the Foundation for Promotion of Cancer Research of Japan.

© Cancer Research Campaign 1998

Table 4 Relative risk of a second primary tumour adjusted for intercurrent smoking, sex, age, performance status, etoposide treatment, radiotherapy and cumulative smoking amount are assessed

| Risk factor | | Relative risk | (95% CI) | P-value |
|---|---|---|---|---|
| Intercurrent smoking | Yes/no | 4.3 | (1.1–15.9) | 0.03 |
| Sex | Female/male | 1.9 | (0.5–6.7) | 0.32 |
| Age (years) | 65 ≤/< 65 | 0.9 | (0.3–3.2) | 0.89 |
| Performance status | 2–4/0–1 | 0.4 | (0.0–3.4) | 0.39 |
| Etoposide | Yes/No | 1.6 | (0.5–5.1) | 0.41 |
| Radiotherapy | Yes/No | 1.6 | (0.4–6.1) | 0.50 |
| Cumulative smoking amount (pack–years) | 45 ≤/< 45 | 0.9 | (0.2–3.3) | 0.82 |

CI, confidence interval.

### REFERENCES

Armstrong J (1990) Long-term outcome of small cell lung cancer. *Cancer Treat Rev* **17**: 1–13

Blum A (1993) Cancer prevention: curtailing the tobacco pandemic. In *Cancer: Principles and Practice of Oncology*, 4th edn, DeVita VT, Hellman S and Rosenberg SA (eds) pp. 480–491. Philadelphia: JB Lippincott

Boice J, Lubin J and Preston D (1991) Epidemiology analysis with a personal computer (EPITOME) NIH. Publication, 91-3180

Heyne KH, Lippman SM, Lee JJ, Lee JS and Hong WK (1992) The incidence of second primary tumors in long-term survivors of small-cell lung cancer. *J Clin Oncol* **10**: 1519–1524

Johnson BE, Matthews MR and Bunn PA (1986) Non-small-cell lung cancer. Major cause of late mortality in patients with small cell lung cancer. *Am J Med* **80**: 1103–1110

Johnson BE, Linnoila RI, Williams JP, Venzon DJ, Okunieff P, Anderson GB and Richardson GE (1995) Risk of second aerodigestive cancers increases in patients who survive free of small-cell lung cancer for more than 2 years. *J Clin Oncol* **13**: 101–111

Osaka Prefectural Department of Environment and Public Health, Osaka Medical Association, Center for Adult Diseases (1993) *Cancer Incidence and Mortality in Osaka 1963–1989*

Osaka Prefectural Department of Health, Osaka Medical Association, Center for Adult Diseases, Osaka (1995) *Annual Report of Osaka Cancer Registry No. 57, Cancer Incidence and Medical Care in Osaka in 1992 and Survival in 1988*

Richardson GE, Tucker MA, Venzon DJ, Linnoila RI, Phelps R, Phares JC, Edison M, Ihde DC and Johnson BE (1993) Smoking cessation after successful treatment of small-cell lung cancer is associated with fewer smoking-related second primary cancers. *Ann Intern Med* **119**: 383–390

SAS Institute (1989) *SAS/STAT User's Guide, Version 6*, Volume 2, 4th edn, pp. 1070–1126. SAS Institute: Cary, NC

Tucker MA, Murray N, Shaw EG, Ettinger DS, Mabry M, Huber MH, Feld R, Shepherd FA, Johnson DH, Grant SC, Aisner J and Johnson BE (1997) Second primary cancers related to smoking and treatment of small-cell lung cancer. *J Natl Cancer Inst* **89**: 1782–1788

© Cancer Research Campaign 1998